# The effect of acupuncture used for cervical spondylosis of vertebral artery type: A protocol for systematic review and meta-analysis

**DOI:** 10.1097/MD.0000000000028956

**Published:** 2022-02-25

**Authors:** Jinxian Lu, Quanmei Song, Yongzheng Zhu, Hongling Jia, Yongchen Zhang

**Affiliations:** a College of Acupuncture and Massage, Shandong University of Traditional Chinese Medicine, Jinan, Shandong, China; b Department of Acupuncture, The Second Affiliated Hospital of Shandong University of Traditional Chinese Medicine, Jinan, Shandong, China.

**Keywords:** acupuncture, cervical spondylosis of vertebral artery type, meta-analysis, protocol

## Abstract

**Background:**

Cervical spondylosis of vertebral artery type is a common clinical disease. With the change of people's lifestyle and the improvement of people who work at desks, its incidence is also increasing, which can seriously affect people's normal life and work. Acupuncture has been gradually accepted and recognized by the public for its green, simple and safe characteristics. In this systematic review, we aimed to evaluate the effect and safety for patients with cervical spondylosis of vertebral artery type to provide evidence for clinical decision making.

**Methods:**

We will search the following 8 databases from their inception to November 2021: Web of Science, the Cochrane Library, PubMed, Embase, Chinese Biomedicine, China National Knowledge Infrastructure, Chinese Scientific Journals Database, and the WanFang Database. All relevant randomized controlled trials (RCTs) that meet the inclusion criteria will be included in our analysis. Literature screening, data extraction and literature quality assessment will be carried out in a step. Data analysis will be performed using RevMan 5.4 software.

**Results:**

Based on the results of this study, we will evaluate the safety and effectiveness of acupuncture in the treatment of vertebral artery type cervical spondylosis.

**Conclusion:**

This study will provide strong evidence-based medical evidence for acupuncture in treating cervical spondylosis of vertebral artery type.

**PROSPERO registration number:**

CRD42021293053.

## 1 Introduction

Cervical spondylosis of vertebral artery type (CSA) is a clinical syndrome in which vertebral-basal artery blood supply is insufficient due to degenerative changes of the cervical spine.^[1,2]^ The clinical manifestations are mainly cervical vertigo, which may be accompanied by a series of symptoms such as dizziness, headache, insomnia, tinnitus, blurred vision, nausea, vomiting, neck and shoulder or occipital neck pain, and even cataplexy.^[3,4]^ Its incidence increases with age,^[5]^ most of which occur in middle-aged and elderly people.^[6]^ In recent years, with the acceleration of people's life rhythm and the increase in the proportion of desk staff, its incidence has increased year by year and tends to be younger. It accounts for about 20% of cervical spondylosis.^[7,8]^

There are many clinical treatment therapies for CSA,^[9]^ with differences and similarities in methods and efficacy. Due to its long course of disease, slow onset and high recurrence rate, treatment is difficult.^[10]^ Western medicine mainly uses nonsurgical treatment and surgical treatment. The high risk and trauma of surgery make conservative treatment the first choice for most patients.^[11]^ Although the former has a certain treatment effect, the long-term effect is poor,^[12]^ adverse reactions may occur, and the recurrence rate is high.^[13]^

Acupuncture, as one of the external treatments of Chinese medicine, has always been valued and recognized by many physicians. Acupuncture has the functions of dredging the meridians, regulating qi and blood. It can not only improve the symptoms of local insufficiency of blood supply and control the course of the disease, but also alleviate the suffering of patients.^[14,15]^ Relevant studies^[16–19]^ have shown that acupuncture can bring vitality to the diseased place by strengthening the body and removing evils, eliminate the adhesion and edema between the local cervical nerves and surrounding tissues, and improve the ischemic and hypoxic state. The clinical effect is significant.^[20]^ Therefore, Acupuncture therapy is widely used in clinical practice, and its treatment methods are diverse. With its advantages of small adverse reaction, good efficacy, quick effect, simple operation and low cost,^[21]^ it demonstrates irreplaceable clinical superiority.^[22]^ However, as a traditional Chinese medicine treatment technique, the biggest problem of acupuncture treatment is the existence of scientific clinical effect certification. Most of the current studies only emphasizes the influence of acupuncture treatment on the hemodynamics of patients, but lacks the clinical efficacy and safety assessment.^[23]^ In order to prove whether acupuncture therapy is really effective for vertebral artery type cervical spondylosis, evidence-based medicine should be adopted to summarize and analyze data with high-quality RCTs, and evaluate its effectiveness and safety, so as to provide evidence for clinical practice.

## 2 Methods

### 2.1 Study registration

The study has been registered in PROSPERO (https://www.crd.york.ac.uk/prospero/). The approved registration number is CRD42021293053. And it was built on the guidelines of Preferred Reporting Items for Systematic Reviews and Meta-Analysis Protocols.^[24]^

### 2.2 Inclusion criteria for study selection

#### 2.2.1 Types of studies

For RCTs on humans, language restrictions were Chinese or English. Reviews, case reports, animal experiments, mechanism studies, data mining, expert clinical experience and nonrandomized controlled trials were excluded.

#### 2.2.2 Types of participants

The included studies should be patients who have been clearly diagnosed with CSA, regardless of gender, race, nationality, course of disease, and severity of disease.

#### 2.2.3 Types of interventions

The experimental group received acupuncture-related therapies (including body acupuncture, scalp acupuncture, electric acupuncture, ear acupuncture, fire acupuncture, warm acupuncture, fast acupuncture, skin acupuncture, moxibustion, cupping, blood pricking, acupoint catgut embedding) or combined with other treatments. No matter the choice of acupoints, the amount of stimulation or the length of treatment. The experimental group could receive the same basic treatment as the control group. The control group received conventional symptomatic treatment, drug treatment, placebo acupuncture, sham acupuncture or no treatment. The control group using acupuncture-related therapies or studies comparing the effects of different acupuncture were excluded.

#### 2.2.4 Types of outcome measures

##### 2.2.4.1 Primary outcomes

The main outcome is the total effective rate. The calculation formula is the number of cases in the group (cured + markedly effective + improved)/total number of cases in the group×100%.

##### 2.2.4.2 Secondary outcomes

Include visual analogue scale, Evaluation Scale for Cervical Vertigo, hemodynamic indexes, clinical symptom evaluation table and adverse reactions.

### 2.3 Data sources

#### 2.3.1 Search strategy

The following databases will be searched from their inception to November 2021: Web of Science, the Cochrane Library, PubMed, Embase, Chinese Biomedicine, China National Knowledge Infrastructure, Chinese Scientific Journals Database, and the WanFang Database. The search strategy used Medical Subject Heading terms and free words and then used Boolean logic operators to connect each search item. In this study, we will take PubMed database (Table 1.) as an example to show the retrieval strategy.

**Table 1:** Search strategy used in PubMed database.

Number	Search terms
#1	acupuncture therapy [MeSH]
#2	acupuncture [Title/Abstract]
#3	acupuncture points[Title/Abstract]
#4	acupuncture treatment[Title/Abstract]
#5	acupuncture,ear[Title/Abstract]
#6	electroacupuncture[Title/Abstract]
#7	fire needle[Title/Abstract]
#8	warm acupuncture[Title/Abstract]
#9	blood-pricking[Title/Abstract]
#10	bloodletting[Title/Abstract]
#11	cupping[Title/Abstract]
#12	fast acupuncture[Title/Abstract]
#13	skin acupuncture[Title/Abstract]
#14	scalp acupuncture[Title/Abstract]
#15	catgut implantation at acupoint[Title/Abstract]
#16	auricular needle[Title/Abstract]
#17	acupuncture-moxibustion[Title/Abstract]
#18	body acupuncture[Title/Abstract]
#19	#1 OR #2 OR #3 OR #4 OR 5 #6 OR #7 OR #8 OR #9 OR #10 OR #11 OR #12 OR #13 OR #14 OR #15 OR #16 OR #17 OR #18
#20	vertebral artery cervical spondylopathy[Title/Abstract]
#21	cervical spondylosis of vertebral artery type[Title/Abstract]
#22	vertebral artery type of cervical spondylosis[Title/Abstract]
#23	cervical spondylotic vertebral arteriopathy[Title/Abstract]
#24	#20 OR #21 OR #22 OR #23
#25	Randomized controlled trial[Publication Type]
#26	placebo[Title/Abstract]
#27	randomized[Title/Abstract]
#28	#25 OR #26 OR #27
#29	#19 AND #24 AND #28

#### 2.3.2 Searching for other resources

We will search for ongoing or unpublished studies from ClinicalTrials.gov, PROSPERO, China Clinical Trials Registry, National Institutes of Health Registry and World Health Organization International Clinical Trials Registry. Manually retrieve any potential gray literature such as conference papers, scientific research results reports and related references.

### 2.4 Data collection and analysis

#### 2.4.1 Selection of studies

**Figure 1:**
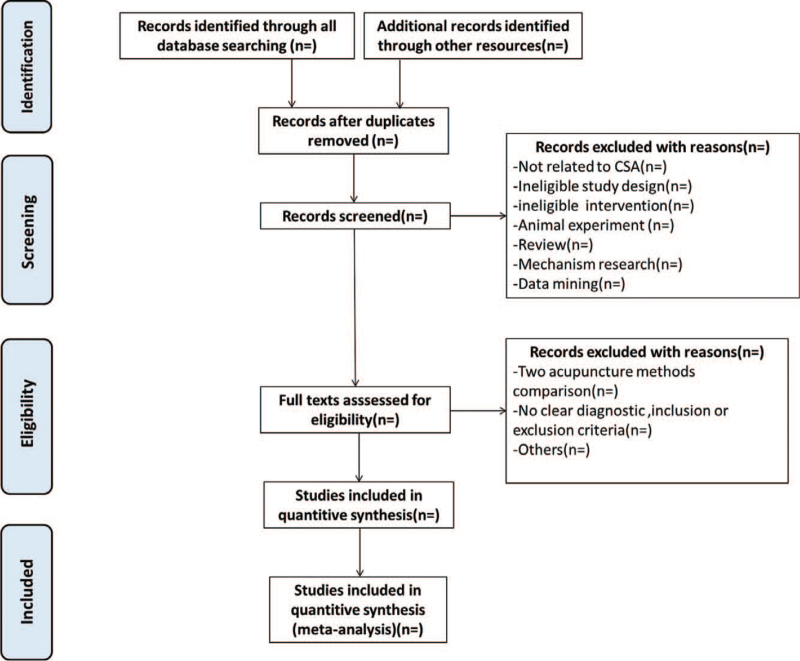
The PRISIMA flow diagram.

All retrieved studies were imported into Endnote X9.1 for management and duplicate documents were removed. Two researchers (JXL and QMS) independently screened the literature according to the inclusion and exclusion criteria. After reading the title and abstract, the studies that did not meet the requirements were deleted. Any problems arising from the screening of the literature will be resolved by the 2 authors through consultation. If the opinions cannot be unified, we will consult and discuss with the third party (YZZ) until consensus is reached. The screening process of this study was shown in the Figure 1.

#### 2.4.2 Data extraction

Regarding the extraction of data, 2 researchers (JXL and QMS) need to independently extract and check with each other. Any disputes should be resolved through consultation with the third party (YZZ) and record the extracted data with the Microsoft Excel 2010 table. The extracted content included the first author, publication year, study type, age, sample size, average course of disease, intervention measures for the treatment group and control group, and the usage and dosage of drugs, course of treatment and outcome indicators.

#### 2.4.3 Dealing with missing data

For missing or ambiguous data, we will contact the corresponding author via email or phone for more detailed content and information. If there is no reply, the relevant data and article will be excluded due to incomplete access.

#### 2.4.4 Risk of bias assessment

Two reviewers (JXL and QMS) independently assessed the quality of RCTs with the “risk of bias assessment tool”^[25]^ recommended by Cochrane Handbook 5.1.0. The Cochrane Risk of Bias Assessment Tool included 7 domains: random sequence generation, allocation concealment, blinding of participants and personnel, blinding of outcome assessment, incomplete outcome data, selective reporting and other bias. They made a judgment of low risk of bias, high risk of bias or unclear risk of bias for each item. Disagreements were resolved through mutual discussion or negotiation with the third party (YZZ).

#### 2.4.5 Statistical analysis

RevMan 5.4 software provided by the Cochrane Collaboration was used for data analysis and synthesis. Choose different measurement indexes according to different data types. Relative risk or odds ratio with 95% confidence intervals was used for dichotomous outcomes, while mean differences or standard mean differences (SMD) with 95% confidence interval was used for continuous outcomes. According to heterogeneity test, fixed effect model or random effect model must be selected for effect size combination. When *P* > .05 or *I*^2^ < 50%, fixed effect model was selected. When *P* < .05 or *I*^2^ > 50%, random effect model was selected. When quantitative synthesis is not applicable, we will separately list the relevant results and report them descriptively.

#### 2.4.6 Sensitivity analysis

We would observe the robustness of the single study to the overall meta-analysis results by eliminating the literature one by one.^[26]^

#### 2.4.7 Subgroup analysis

We will conduct subgroup analysis according to the following groups to explore the causes of heterogeneity.

1. Length of treatment: The first group is 2 weeks, the second group is 4 weeks.
2. Oral western medicine used in the treatment.

#### 2.4.8 Assessment of reporting biases

If more than 10 studies are finally included, we will use the funnel plot drawn by Revman 5.4 software to test whether the publication bias has occurred.

#### 2.4.9 Quality of evidence

The quality of evidence will be evaluated through the Grading of Recommendations Assessment, Development and Evaluation approach.^[27]^ There are 4 levels of evidence: very low, low, medium or high.

## 3 Discussion

CSA involves a series of syndromes caused by the stimulation and compression of cervical nerve roots, spinal cord, vertebral arteries, and cervical sympathetic nerves.^[28]^ Its pathogenesis is complex, and some of the pathological mechanisms are not yet fully understood. Vasospasm caused by mechanical compression and vertebral artery sympathetic nerve stimulation is currently recognized and the 2 main pathogenic factors influence each other or cause each other.^[29]^ But it is undeniable that acupuncture has played an important role in reducing symptoms and improving hemodynamics, which can better relieve the discomfort of the patient's head and neck.

High-quality meta-analyses/systematic evaluations are one of the important sources of evidence-based medicine for obtaining the best evidence and one of the best bases for clinical decision making in acupuncture.^[30]^ The conclusions of this study can provide guidance for clinical acupuncture treatment of vertebral artery type cervical spondylosis. Only by focusing on the quality of methodology and the collection of large-sample, multi-center high-quality RCTs, can the reliability and accuracy of meta-analysis conclusions be better improved and the safety and effectiveness of acupuncture treatment be objectively evaluated.

## Author contributions

**Investigation:** Jinxian Lu.

**Methodology:** Jinxian Lu, Quanmei Song, Yongzheng Zhu.

**Resources:** Jinxian Lu, Yongzheng Zhu, Hongling Jia.

**Software:** Jinxian Lu, Quanmei Song.

**Supervision:** Hongling Jia, Yongchen Zhang.

**Validation:** Quanmei Song, Yongzheng Zhu.

**Writing – original draft:** Jinxian Lu.

**Writing – review & editing:** Yongchen Zhang.
